# Development of machine learning-based predictive models for fertility intentions in patients with Crohn's disease

**DOI:** 10.3389/frph.2026.1836350

**Published:** 2026-05-22

**Authors:** Jinghan Liu, Jinli Bu, Xiao Han, Zhen Wang, Danhong Dong, Meihao Wei, Qiaoyu Wu

**Affiliations:** Nursing Department, Sir Run Run Shaw Hospital, Zhejiang University School of Medicine, Hangzhou, China

**Keywords:** Crohn's disease, fertility intention, machine learning, psychosocial factors, SHAP

## Abstract

**Background:**

Fertility intentions in patients with Crohn's disease (CD) are shaped by complex interactions between biological and psychosocial factors. However, predictive tools that integrate these dimensions remain underdeveloped.

**Objective:**

This study aimed to construct and validate interpretable machine learning (ML) models to predict fertility intentions among reproductive-age patients with CD, and to identify key psychosocial determinants driving reproductive decision-making.

**Methods:**

A total of 276 CD patients aged 18–45 years were prospectively recruited from a tertiary center in China. Participants completed standardized assessments covering demographic, clinical, psychosocial, and fertility-related variables. Fertility intention was categorized based onself-reported desire and timing of childbearing. Eight ML models, including neural networks and extreme gradient boosting (XGBoost), were trained on 80% of the data and tested on the remaining 20%. Model performance was evaluated using area under the receiver operating characteristic curve (AUC), accuracy, calibration, and decision curve analysis. SHapley Additive exPlanations (SHAP) were applied for model interpretability.

**Results:**

Among the eight algorithms, neural networks and XGBoost demonstrated the highest discrimination (AUC = 0.718 and 0.710, respectively). SHAP analysis revealed that marital status, actual desired number of children, and perceived family support were the most influential predictors of fertility intention. Multivariable logistic regression confirmed marital status and reproductive preference as independent correlates (*p* < 0.05). These models achieved favorable calibration and clinical utility, as demonstrated by decision curve analysis.

**Conclusions:**

This study presents a novel application of interpretable ML models for predicting fertility intentions in CD patients. The findings highlight the dominant role of relational and psychosocial factors over disease activity, offering a methodological and clinical framework for integrating fertility counseling into inflammatory bowel disease (IBD) care. Incorporating structured psychosocial assessment may enable more personalized and anticipatory reproductive support in young patients with CD.

## 1 Background

Crohn's disease (CD) is a chronic inflammatory condition with an unclear etiology, classified alongside ulcerative colitis (UC) under the umbrella of inflammatory bowel disease (IBD) (1, 2). The primary clinical manifestations of CD include diarrhea, abdominal pain, and weight loss. Compared to UC, CD can affect the entire gastrointestinal tract and is more likely to cause fatigue, fever, recurrent fistula formation, perianal abscesses or fistulas, and extra-intestinal manifestations. In China, the incidence of CD has risen sharply over the past two decades alongside rapid economic development and lifestyle westernization, now ranking among the highest in Asia (3, 4). Although there is no definitive cure for CD, it is manageable and requires long-term maintenance therapy. In addition to the heavy economic and psychological burden it imposes, along with profound effects on academic, career, and marital life, concerns regarding the potential reproductive toxicity of certain medications further compound the dilemmas many patients face when making reproductive decisions (5).

The World Health Organization (WHO) defines the reproductive age range as 15 to 49 years old (WHO) (45). The peak onset of CD occurs between 18 and 35 years of age, coinciding precisely with the optimal reproductive window. Consequently, many young patients have not yet started a family or completed their reproductive plans when diagnosed. Although fertility is generally unaffected in quiescent CD (6), it may be transiently reduced during active flares. This is attributable not only to malnutrition but also to the systemic inflammatory milieu, which can disrupt the hypothalamic–pituitary–gonadal axis, impair oocyte quality via pro-inflammatory cytokines, and reduce endometrial receptivity (7, 8). More importantly, the dilemma surrounding reproductive decisions is largely driven by voluntary factors, including fear of disease worsening, concerns about drug-related and maternal–fetal risks, and insufficient disease-specific knowledge (9, 10). As a result, some patients may miss the optimal fertility window or forgo childbearing altogether. Indeed, approximately 75% of patients with CD express concerns about passing the disease to their offspring, 30% choose not to have children, and 17% remain voluntarily childless. The incidence of these concerns is significantly higher compared to the general population (11–13). Given the diverse clinical manifestations and prognostic features of CD, patients often face unique reproductive needs. Therefore, fertility intentions and family planning have become an important issue in the care of young CD patients; however, research on the factors influencing these intentions remains limited.

Fertility intention is a multidimensional construct encompassing the ideal, desired, and intended number of children, shaped by individual attitudes, partner preferences, and broader social and policy contexts (14–16). Existing studies further indicate that the fertility intentions of chronic disease patients are influenced by factors such as disease severity (5, 17–19), drug treatment (5), reproductive health knowledge (12), psychological state (19–21), and socio-cultural background (22, 23). However, there is still a limited exploration of fertility intentions in CD patients.

In recent years, machine learning (ML) has demonstrated significant advantages over traditional statistical methods (e.g., logistic regression) for modeling complex, multifactorial outcomes in biomedical research. ML algorithms are not constrained by linear assumptions and can effectively capture high-dimensional, nonlinear interactions among variables, making them particularly suited for exploring intricate behavioral and psychosocial phenomena (24, 25). Furthermore, the application of explainable AI techniques, such as Shapley Additive exPlanations (SHAP), addresses the “black-box” limitation of advanced models by providing transparent, individual-level interpretations of predictive contributions (26, 27). This capability is crucial for building clinician trust and facilitating the translation of predictive tools into actionable, personalized care strategies (28). While ML has been successfully applied to predict various clinical and behavioral outcomes, including suicide risk (29) and cognitive impairment (30), its potential to elucidate and predict fertility intentions—a complex decision-making process at the intersection of health, psychology, and social context—remains largely untapped, especially within the IBD population.

Therefore, this study aims to bridge this gap by developing and validating interpretable ML models to predict fertility intentions in patients with CD. We hypothesize that ML models can integrate multidimensional clinical and psychosocial data to achieve superior predictive accuracy compared to conventional methods, and that explainability frameworks will identify key, modifiable drivers of reproductive decision-making. The findings are expected to provide a novel, data-driven framework to guide the integration of personalized fertility counseling and psychosocial support into the routine care of young adults with CD.

## 2 Methods

### 2.1 Participants and recruitment

Participants diagnosed with CD in the Department of Gastroenterology at Sir Run Run Shaw Hospital, Zhejiang University, between December 2024 and June 2025 were recruited for this study. Participants were invited to join the study and complete a questionnaire during their hospitalization. Eligible participants were those who met the following criteria: aged 18–45 years, able to read and write in Chinese, and diagnosed with CD. Inclusion Criteria: ① Patients diagnosed with CD or ulcerative colitis based on the IBD diagnostic criteria established by the IBD Group of the Chinese Society of Gastroenterology in 2023 [4–5]; ② Diagnosis confirmed for more than 3 months;③ Patients aged 18–49 years who have not yet had children; ④ Voluntarily participating in the interview.

Exclusion criteria were as follows: (1) infertility attributed to irreversible congenital or acquired conditions unrelated to Crohn's disease (e.g., congenital uterine absence, bilateral oophorectomy, or known genetic sterility); (2) current pregnancy or breastfeeding at the time of enrollment; (3) severe psychiatric or cognitive disorders that would preclude informed consent or valid completion of the questionnaires; (4) any other severe, life-limiting comorbidity (e.g., end-stage malignancy) that would dominate the patient's reproductive decision-making independently of CD.

### 2.2 Measurements

#### 2.2.1 Demographic information

The following variables were collected from the participants: age, gender, educational level, marital status, childbearing experiences, monthly income, medical insurance, current work or study status, disease duration, whether using biologics, whether using immunosuppressants, IBD-related surgical treatment, number of chronic diseases, disease activity, ideal number of children, ideal gender of children, actual expected number of children, actual expected gender of children, fertility policy, sense of coherence, social support, and disease acceptance.

#### 2.2.2 Fertility intentions

In accordance with the widely accepted tripartite classification of fertility intention in academic literature, this study adopts three commonly operationalized dimensions: intended timing of childbirth, desired number of children, and preferred gender of children was primarily assessed through participants' planned timing for childbearing. Specifically, participants were asked: “Do you intend to have children?” The response options included: “Definitely will have children”, “Intend to, depending on future circumstances”, “Probably will not have children”, and “Definitely will not have children”. For data analysis purposes, participants selecting either “Definitely will have children” or “Intend to, depending on future circumstances” were classified as having positive fertility intentions. Those selecting “Probably will not have children” or “Definitely will not have children” were categorized as having no fertility intention.

The desired number of children was assessed through both ideal and expected fertility preferences. Participants were asked: “Disregarding practical considerations (such as policy, financial constraints, or health status), how many children do you think is ideal for a family?” “Given your current circumstances, how many children do you realistically expect to have?” Response options for both questions were:“0, 1, 2, 3 or more, and Undecided”. Preferred gender of children was evaluated through questions regarding both ideal and expected gender preference. Participants were asked to indicate their preference by selecting from: “Boy”, “Girl”, “No preference”, or “Undecided”.

Although fertility intention (dichotomized as present or absent based on planned childbearing) and reproductive preferences (desired number and gender of children) are conceptually related, they were treated as distinct constructs in this study. The former represents a global motivational state, while the latter reflect specific, value-laden dimensions of fertility ideation that may further modulate the strength of intention. All participants, irrespective of their fertility intention, were asked to report these preferences, and the responses were included as potential predictors of the binary outcome.

#### 2.2.3 Multidimensional scale of perceived social support (MSPSS)

Social support was measured using the Multidimensional Scale of Perceived Social Support (MSPSS), developed by Zimet et al. (31). This scale aims to assess an individual's perception of the adequacy of social support from three sources: family (items 3, 4, 8, and 11), friends (items 6, 7, 9, and 12), and significant others (items 1, 2, 5, and 10). Respondents rate each item using a seven-point Likert scale, where 1 represents “strongly disagree” and 7 represents “strongly agree”. The total score ranges from 12 to 84, with higher scores indicating stronger perceived social support. The MSPSS demonstrates good internal consistency, with a Cronbach's *α* of 0.95, indicating high reliability (31).

#### 2.2.4 The simplified CD activity index (CDAI)

The Simplified CD Activity Index (CDAI), also known as the Harvey–Bradshaw Index (HBI), is commonly used to assess clinical activity in CD patients. The index includes five components: overall health, abdominal pain, diarrhea, abdominal mass, and associated complications. It is based on the patient's symptoms over the past seven days. Scores from 0 to 4 indicate disease remission, scores from 5 to 8 indicate moderate disease activity, and scores of 9 or higher indicate severe disease activity.

#### 2.2.5 Assessment of sense of coherence

The Sense of Coherence (SOC) was assessed using the 13-item self-report scale (SOC-13), originally developed by Aaron Antonovsky to measure an individual's global orientation toward life stressors and capacity to maintain health and well-being (32). The scale captures three interconnected dimensions: comprehensibility, manageability, and meaningfulness. Respondents rate each item on a 7-point Likert scale ranging from 1 (“Very seldom or never”) to 7 (“Very often”). After reverse-coding five negatively phrased items, a total score was computed by summing all responses, yielding a potential range of 13 to 91. Higher total scores indicate a stronger SOC, reflecting greater resilience and adaptive capacity in the face of life demands, including those imposed by chronic illness. The Chinese version of the SOC-13 has been validated in previous studies and demonstrated good internal consistency in the present sample (Cronbach's α = 0.85) (33).

### 2.3 Data collection

This study utilized the specialized online survey platform Wenjuanxing to collect data through questionnaires. The sample was selected from patients diagnosed with or hospitalized for CD at a tertiary hospital in Zhejiang data to the Wenjuanxing platform, generating a unique survey link. This link was then shared with registered participants for them to complete the questionnaire online. To avoid duplicate submissions and ensure traceability of data, each participant was assigned a unique identifier, which could only be used once. Additionally, mandatory response fields were set, requiring participants to complete all questions before submission, ensuring the integrity of the data. For registered participants who were unable to complete the questionnaire online, one-on-one interviews were conducted through both written and verbal communication. Upon completion of data collection, all survey responses were systematically organized and subjected to preliminary checks. Data quality control—exclusion of invalid questionnaires. To ensure data integrity, strict quality-control criteria were applied to remove invalid responses from the analytical dataset: questionnaires in which the same response option was selected for all items (indicating likely random or non-engaged answering), responses that did not follow instructions, or those containing logical or formatting inconsistencies were excluded.

### 2.4 Statistical analysis

To ensure the quality and usability of variables, data preprocessing was first conducted. Consistent with common methodological practice in clinical prediction modeling, a pre-specified threshold of 20% missing values was used to determine variable exclusion to ensure the stability of subsequent imputation and modeling (34–36). In our dataset, the maximum missing rate of all collected variables was 5.434%, specifically: CDAI abdominal pain (0.724%), CDAI abdominal mass (0.362%), Ideal number of children (5.434%), Ideal gender of children (5.434%), Actual desired number of children (5.434%), Actual desired gender of children (5.434%), and Influenced by fertility policies (5.434%). The remaining variables had complete data. For the variables retained, missing data were imputed using the Multivariate Imputation by Chained Equations (MICE) method. Summary statistics were calculated using the original, non-imputed data: normally distributed continuous variables were expressed as mean ± standard deviation, whereas non-normally distributed continuous variables were expressed as median with interquartile range (25th–75th percentile). Categorical variables were expressed as counts and percentages. Using the original data for descriptive statistics ensures that the reported distributions reflect the actual observed values before imputation, while imputation was applied only for subsequent modeling to avoid bias due to missingness. Independent t test and one-way ANOVA were used for comparison between groups. The dataset was then randomly split into a training set (80%) and a testing set (20%). The training set was used for model development, while the testing set was reserved for model evaluation and interpretation.

To mitigate the risk of model overfitting, we employed a 10-fold cross-validation strategy for hyperparameter tuning. This approach partitions the training data into 10 subsets and performs iterative cross-validation. Given the potential multicollinearity among predictors in cross-sectional data, we applied the Least Absolute Shrinkage and Selection Operator (LASSO) regression for variable selection. LASSO addresses linear relationships among variables by applying a penalty that shrinks the coefficients of irrelevant predictors to zero, thereby retaining only statistically robust variables and enhancing model interpretability (30, 37). It is important to clarify that in this study, LASSO served solely as a model-agnostic feature selection tool, not as a modeling assumption that constrains subsequent algorithms. The objective of applying LASSO prior to model development was threefold: (1) to reduce the dimensionality of the feature space and minimize the risk of overfitting given our moderate sample size; (2) to establish a uniform predictor set across all eight candidate models, ensuring that differences in model performance could be attributed to algorithmic capacity rather than differential access to features; and (3) to enhance clinical interpretability by retaining only those variables with demonstrable statistical relevance. Critically, the linearity assumption of LASSO applies only to the feature selection stage and does not impose any linear constraint on the downstream models. Once a parsimonious predictor set was identified, it was then passed to all models—including highly flexible non-linear algorithms such as neural networks and tree-based ensembles—which were free to capture complex interactions and non-linear relationships during training.

Following LASSO-based variable selection, we developed a total of eight ML models: logistic regression, neural network, support vector machine (SVM), extreme gradient boosting (XGBoost), k-nearest neighbors (KNN), decision tree, random forest, and Light Gradient Boosting Machine (LightGBM). These models were chosen to represent a diverse set of ML paradigms, encompassing linear classifiers, kernel-based methods, individual learners, ensemble approaches, and gradient-boosted frameworks—all well-suited for identifying predictors of fertility intention in cross-sectional data.

#### 2.4.1 Hyperparameter configuration and model specification

To ensure full reproducibility, the final model architectures and hyperparameters were as follows. Hyperparameters were tuned via grid search with 10-fold cross-validation (or 5-fold for LightGBM) on the training set, using AUC as the primary optimization metric unless otherwise specified.

Logistic Regression: A standard multivariable logistic regression model was fitted via maximum likelihood using glm with a binomial family and logit link; no additional regularization was applied.

KNN: The kknn package was employed with a triangular kernel and Euclidean distance. The number of neighbors (k) was selected from 1 to 30 by maximizing the AUC on the test set. The final model used k = 28 and kernel = “triangular”.

Decision Tree: A classification and regression tree (CART) was built with the rpart package. The complexity parameter (cp) was optimized over [0.001, 0.3] (step 0.001) using 10-fold cross-validation. The final tree used cp = 0.231, with other parameters kept at rpart defaults (minsplit = 20, minbucket = 7, maxdepth = 30).

Random Forest: The randomForest package was used. The number of variables randomly sampled at each split (mtry) was tuned from 1 to $\sqrt{P}$ via 10-fold cross-validation in caret, yielding an optimal mtry = 2. The number of trees (ntree) was then selected to minimize the out-of-bag error rate over 50–1,000 (step 50), resulting in ntree = 300.

XGBoost: The xgboost package was used with objective “binary:logistic” and evaluation metric “auc”. A grid search was performed over max_depth ∈ {2,3,4,5}, eta ∈ {0.01,0.1,0.2}, and nrounds ∈ {50,100,150}. The best combination was max_depth = 2, eta = 0.01, and the model was trained for 150 boosting rounds with base_score = 0.5.

LightGBM: The lightgbm package was used. A grid search was conducted over num_leaves ∈ {15,31}, max_depth ∈ {−1,1,3}, learning_rate ∈ {0.1,0.2}, n_estimators = 50, min_data_in_leaf = 30, and L1/L2 regularization [λ_l1, λ_l2 ∈ (0,1)]. The combination maximizing 5-fold cross-validated AUC was selected: objective = “binary”, metric = “auc”, learning_rate = 0.1, num_leaves = 15, max_depth = −1 (no limit), n_estimators = 50, min_data_in_leaf = 30, λ_l1 = 0, and λ_l2 = 0.

SVM: A support vector classifier with a radial basis function (RBF) kernel was tuned using tune.svm (10-fold cross-validation). The optimal hyperparameters were gamma = 0.01 and cost = 10. Probability estimation was enabled.

Neural Network: A feed forward neural network was built with the neuralnet package. Six hidden-layer configurations were tested: single hidden layers with 2, 3, 4, or 5 neurons, and two-hidden-layer structures (2,1) and (2,2). All hidden neurons used the logistic (sigmoid) activation function; the output layer used a single neuron with sigmoid activation (linear.output = FALSE). The architecture achieving the highest test AUC (0.718) was a two-hidden-layer network with 2 neurons in the first hidden layer and 1 neuron in the second (i.e., structure 2 → 1).

A complete summary of all hyperparameters is provided in Supplementary Table S1.

Model performance was evaluated on both training and testing datasets using a range of metrics: accuracy, precision, recall, F1 score (the harmonic mean of precision and recall), specificity, sensitivity, the receiver operating characteristic (ROC) curve, and the area under the ROC curve (AUC). Calibration of models was assessed using calibration plots and the Brier score. Additionally, decision curve analysis (DCA) was employed to examine the clinical utility of each model. Differences in AUC values between models were tested using DeLong's test. To minimize random error, all evaluation metrics were calculated using 10-fold cross-validation (38).

#### 2.4.2 Selection of models for SHAP interpretation

Among the eight algorithms, the neural network achieved the highest discriminative performance on the test set (AUC = 0.718), while the extreme gradient boosting (XGBoost) model ranked second (AUC = 0.710). To balance maximal predictive accuracy with clinical plausibility, both models were retained for SHAP-based interpretation. The neural network was chosen because of its superior AUC; XGBoost was additionally selected because its top predictors showed strong concordance with the independent multivariable logistic regression analysis, thereby providing a complementary, more clinically familiar validation of the importance patterns identified by the neural network. Presenting both models thus offers a more robust and trustworthy depiction of the determinants of fertility intention.

To enhance the interpretability of the models, we employed SHAP to evaluate variable importance and assess how individual features influenced fertility intentions among patients. SHAP is a game-theoretic approach based on Shapley values, offering consistent and locally accurate attributions of model predictions to feature contributions.

All data analyses were conducted using R software (version 4.4.1). The following R packages were utilized: foreign, dplyr, tidyverse, compareGroups, gtsummary, pbapply, DALEX, caret, ggplot2, pROC, ResourceSelection, DynNom, survey, plotROC, randomForest, xgboost, lightgbm, kknn, neuralnet, NeuralNetTools, gridExtra, partykit, and missForest.

## 3 Results

### 3.1 Patient characteristics

A total of 276 patients with CD were enrolled in this study, among whom 110 individuals (39.9%) expressed a desire to have children. The cohort consisted of 218 males (79%) and 58 females (21%). The median age of participants was 28 years (IQR: 23–34), and the median body mass index (BMI) was 21.49 kg/m^2^ (IQR: 19.16–24.54). A majority of patients (229, 83%) had attained a university-level education or higher. Detailed characteristics are provided in Table 1.

**Table 1:** Baseline characteristics of participants with Crohn's disease based on fertility intention.

Variable	Total (*n* = 276)	Fertility intention		*P* value
		No (*n* = 166)	Yes (*n* = 110)	
Age, Median (IQR)	28.00 [23.00–34.00]	26.00 [22.00–32.00]	31.00 [26.00–35.00]	<0.001
Gender, *n* (%)				0.214
Male	218 (79%)	127 (77%)	91 (83%)	
Female	58 (21%)	39 (23%)	19 (17%)	
BMI, kg/m²,Median (IQR)	21.49 [19.16–24.54]	21.33 [19.05–24.22]	22.12 [19.37–25.26]	0.121
Education level, *n* (%)				0.372
High school or below	47 (17%)	31 (19%)	16 (15%)	
University or above	229 (83%)	135 (81%)	94 (85%)	
Marital status, *n* (%)				<0.001
Never married	164 (59%)	123 (74%)	41 (37%)	
Married	106 (38%)	39 (23%)	67 (61%)	
Divorced	4 (1.4%)	3 (1.8%)	1 (0.9%)	
Widowed	1 (0.4%)	0 (0%)	1 (0.9%)	
Other	1 (0.4%)	1 (0.6%)	0 (0%)	
Monthly income, RMB, *n* (%)				0.333
<5,000	40 (14%)	28 (17%)	12 (11%)	
5,000–10,000	123 (45%)	74 (45%)	49 (45%)	
>10,000	113 (41%)	64 (39%)	49 (45%)	
Disease duration, years, *n* (%)				0.218
<5	182 (66%)	116 (70%)	66 (60%)	
5–10	66 (24%)	36 (22%)	30 (27%)	
>10	28 (10%)	14 (8.4%)	14 (13%)	
Biologic biosimilar therapies, *n* (%)			0.400	
Yes	251 (91%)	149 (90%)	102 (93%)	
No	25 (9.1%)	17 (10%)	8 (7.3%)	
Immunosuppressants, *n* (%)				0.838
Yes	61 (22%)	36 (22%)	25 (23%)	
No	215 (78%)	130 (78%)	85 (77%)	
Surgical treatment for IBD, *n* (%)				0.882
Yes	137 (50%)	83 (50%)	54 (49%)	
No	139 (50%)	83 (50%)	56 (51%)	
The number of chronic diseases, *n* (%)			0.732	
0	161 (58%)	100 (60%)	61 (55%)	
1	101 (37%)	58 (35%)	43 (39%)	
≥2	14 (5.1%)	8 (4.8%)	6 (5.5%)	
CDAI scores, Median (IQR)	5.00 [3.00–6.00]	5.00 [3.00–7.00]	5.00 [3.00–6.00]	0.962
Ideal number of children, *n* (%)				0.003
0	8 (3.1%)	8 (5.3%)	0 (0%)	
1	87 (33%)	51 (34%)	36 (33%)	
2	132 (51%)	67 (44%)	65 (59%)	
3 or more	3 (1.1%)	1 (0.7%)	2 (1.8%)	
Other	31 (12%)	24 (16%)	7 (6.4%)	
Ideal gender of children, *n* (%)				0.020
Male	10 (3.8%)	5 (3.3%)	5 (4.5%)	
Female	48 (18%)	30 (20%)	18 (16%)	
Both	123 (47%)	60 (40%)	63 (57%)	
Other	80 (31%)	56 (37%)	24 (22%)	
Actual desired number of children, *n* (%)			<0.001	
0	20 (7.7%)	20 (13%)	0 (0%)	
1	126 (48%)	63 (42%)	63 (57%)	
2	65 (25%)	25 (17%)	40 (36%)	
Other	50 (19%)	43 (28%)	7 (6.4%)	
Actual desired gender of children, *n* (%)			0.029	
Male	12 (4.6%)	7 (4.6%)	5 (4.5%)	
Female	63 (24%)	36 (24%)	27 (25%)	
Both	88 (34%)	41 (27%)	47 (43%)	
Other	98 (38%)	67 (44%)	31 (28%)	
Influenced by fertility policies, *n* (%)			0.641	
Yes	181 (69%)	103 (68%)	78 (71%)	
No	80 (31%)	48 (32%)	32 (29%)	
Family Support, Median (IQR)	23.00 [18.00–26.00]	21.00 [16.00–25.00]	24.00 [21.00–27.00]	<0.001
Friend Support, Median (IQR)	20.00 [16.00–24.00]	20.00 [16.00–24.00]	21.50 [17.00–24.00]	0.024
Other Support, Median (IQR)	20.00 [16.00–24.00]	19.00 [16.00–23.00]	22.00 [17.00–25.00]	0.001
Comprehensibility, Median (IQR)	20.00 [15.00–23.00]	21.00 [16.00–23.00]	18.00 [14.00–22.00]	0.029
Manageability, Median (IQR)	18.00 [12.00–21.00]	19.00 [13.00–21.00]	16.00 [11.00–20.00]	0.033
Meaningfulness, Median (IQR)	17.00 [15.00–19.00]	17.00 [15.00–19.00]	17.00 [16.00–19.00]	0.102
Total Social Support, Median (IQR)	62.00 [52.00–72.00]	60.00 [49.00–70.00]	67.00 [57.00–74.00]	<0.001

The continuous variables were presented as median (interquartile range, IQR), and the categorical variables were shown as number and percentages. BMI, body mass index; SD, standard deviation.

Compared to patients without fertility intention, those with fertility intention were significantly older, had a higher likelihood of being married, were more accepting of either gender for future children, expressed a desire for a greater number of children, and reported higher levels of support from family, friends, and other sources. However, they exhibited significantly lower scores in the Comprehensibility and Manageability domains of the SOC scale. All of these differences reached statistical significance.

### 3.2 Data preprocessing and variables selection

**Figure 1:**
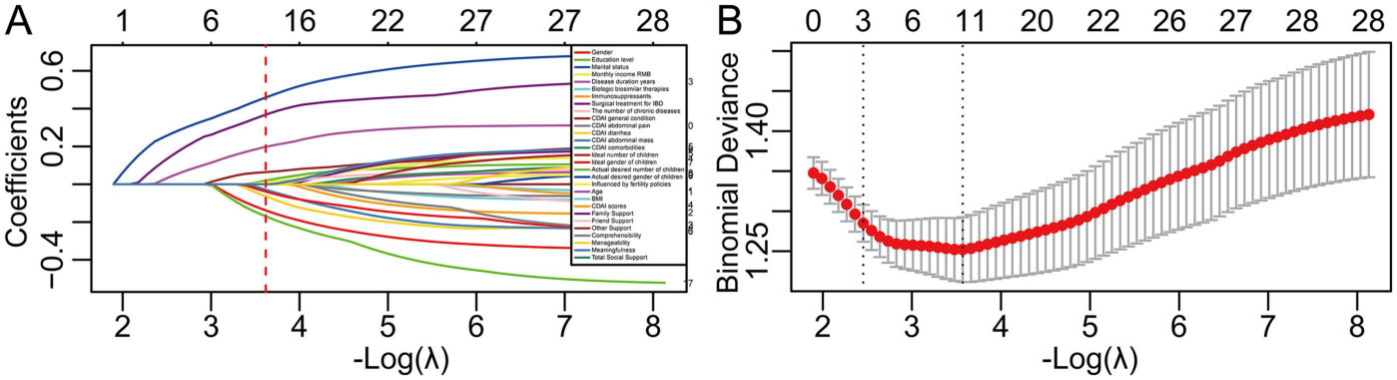
Identification of potential predictors through LASSO regression. **(A)** The plot showing the process of variable selection in the Lasso regression model. **(B)** The optimal value of the regularization parameter *λ* in the Lasso regression model determined using ten-fold cross-validation.

We randomly divided the dataset into a training set (*n* = 221, 80%) and a testing set (*n* = 55,20%). There were no significant differences in candidate variables between the two groups (*p* > 0.05). Key variables were selected using LASSO regression. At log(λ) = −3.478, the mean squared error (MSE) reached its minimum. The variables retained in the final model included: gender, education level, marital status, monthly income, CDAI diarrhea, CDAI abdominal mass, ideal gender of children, actual desired number of children, age, family support, and other support (Figure 1).

### 3.3 Model performance

**Figure 2:**
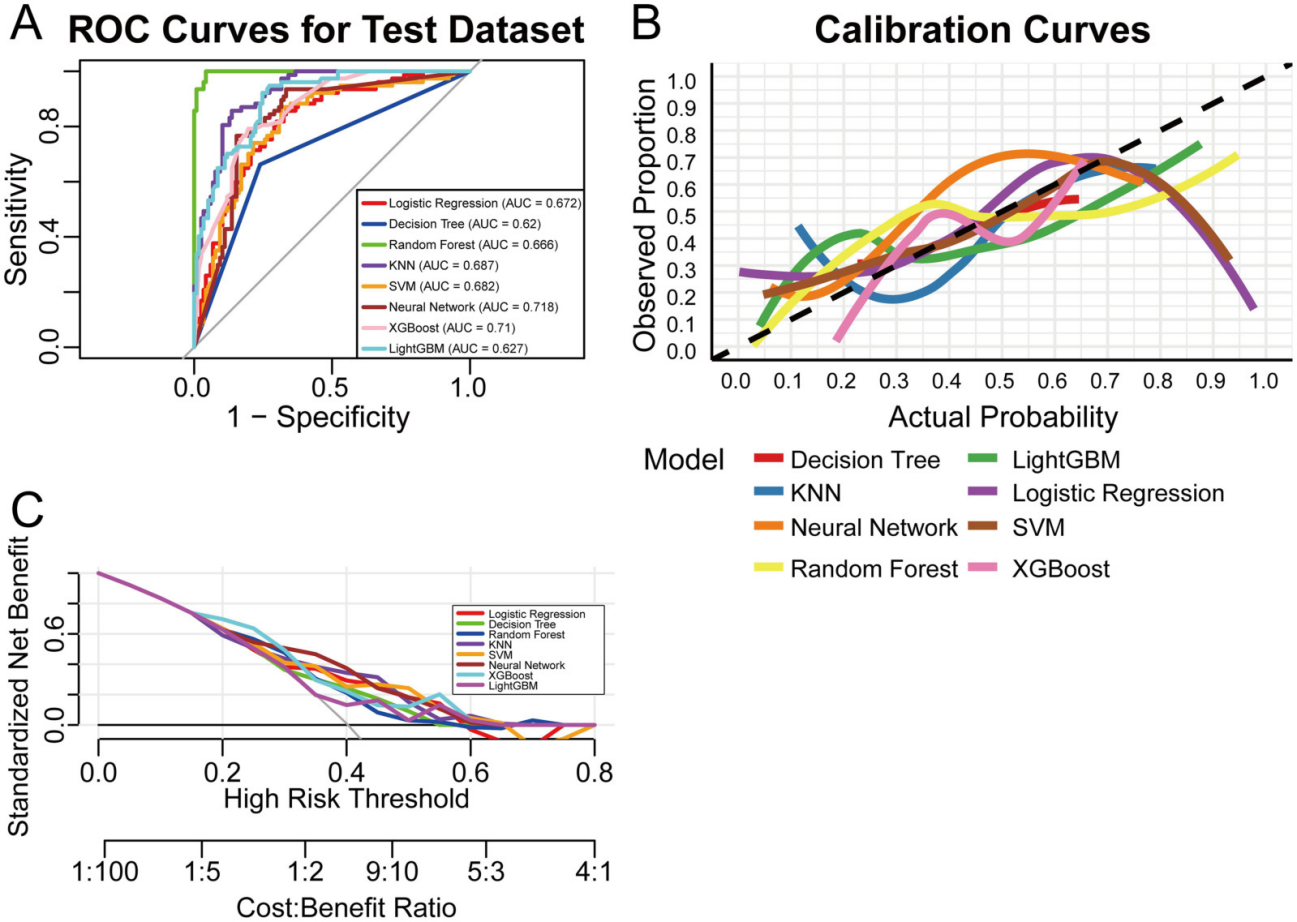
Performance and comparison of eight different predictive models. **(A)** ROC curve for the test set. **(B)** Calibration curve for the test set. **(C)** Decision curve analysis for the test set. SVM, support vector machine; XGBoost, extreme gradient boosting; KNN, k-nearest neighbors; LightGBM, Light Gradient Boosting Machine.

We constructed eight ML models and optimized their hyperparameters through grid search. Table 2 and Figure 2 illustrate the specific performance of each model in the test set. The neural network model demonstrated the best performance, achieving an AUC of 0.718, an accuracy of 0.671, and a precision of 0.607. This was followed by the XGBoost model, which attained an AUC of 0.710 and an accuracy of 0.610 (Figure 2A). These models exhibited moderate discriminative ability in distinguishing reproductive intention among patients with CD. In terms of calibration, the neural network model closely aligned with the diagonal in the low-probability region but deviated slightly in the high-probability region, reflecting its reasonable sensitivity in predicting actual probabilities of reproductive intention. A similar trend was observed in the calibration of the XGBoost model (Figure 2B). DCA further supported these findings, indicating that both the neural network and XGBoost models provided favorable net benefits for clinical decision-making across most threshold ranges (Figure 2C).

**Table 2:** The predictive performance of different machine learning models.

Model	Features	AUC	Accuracy	Precision	Recall	F1-score
Train datast						
LR	11	0.816	0.753	0.730	0.597	0.657
KNN	11	0.921	0.851	0.800	0.831	0.815
DT	11	0.712	0.722	0.646	0.662	0.654
RF	11	0.997	0.969	0.986	0.935	0.96
XGBoost	11	0.861	0.784	0.701	0.792	0.745
LightGBM	11	0.898	0.804	0.791	0.688	0.736
SVM	11	0.811	0.753	0.710	0.636	0.672
Nnet	11	0.828	0.814	0.766	0.766	0.766
Test datast						
LR	11	0.672	0.683	0.640	0.485	0.553
KNN	11	0.687	0.695	0.618	0.636	0.627
DT	11	0.62	0.634	0.546	0.546	0.546
RF	11	0.666	0.573	0.464	0.394	0.426
XGBoost	11	0.71	0.61	0.514	0.546	0.53
LightGBM	11	0.627	0.634	0.552	0.485	0.516
SVM	11	0.682	0.695	0.667	0.485	0.558
Nnet	11	0.718	0.671	0.607	0.515	0.557

LR, logistic regression; Nnet, neural network; SVM, support vector machine; XGBoost, extreme gradient boosting; KNN, k-nearest neighbors; DT, decision tree; RF, random forest; LightGBM, light gradient boosting machine; AUC, the area under the ROC curve.

### 3.4 Model interpretation

**Figure 3:**
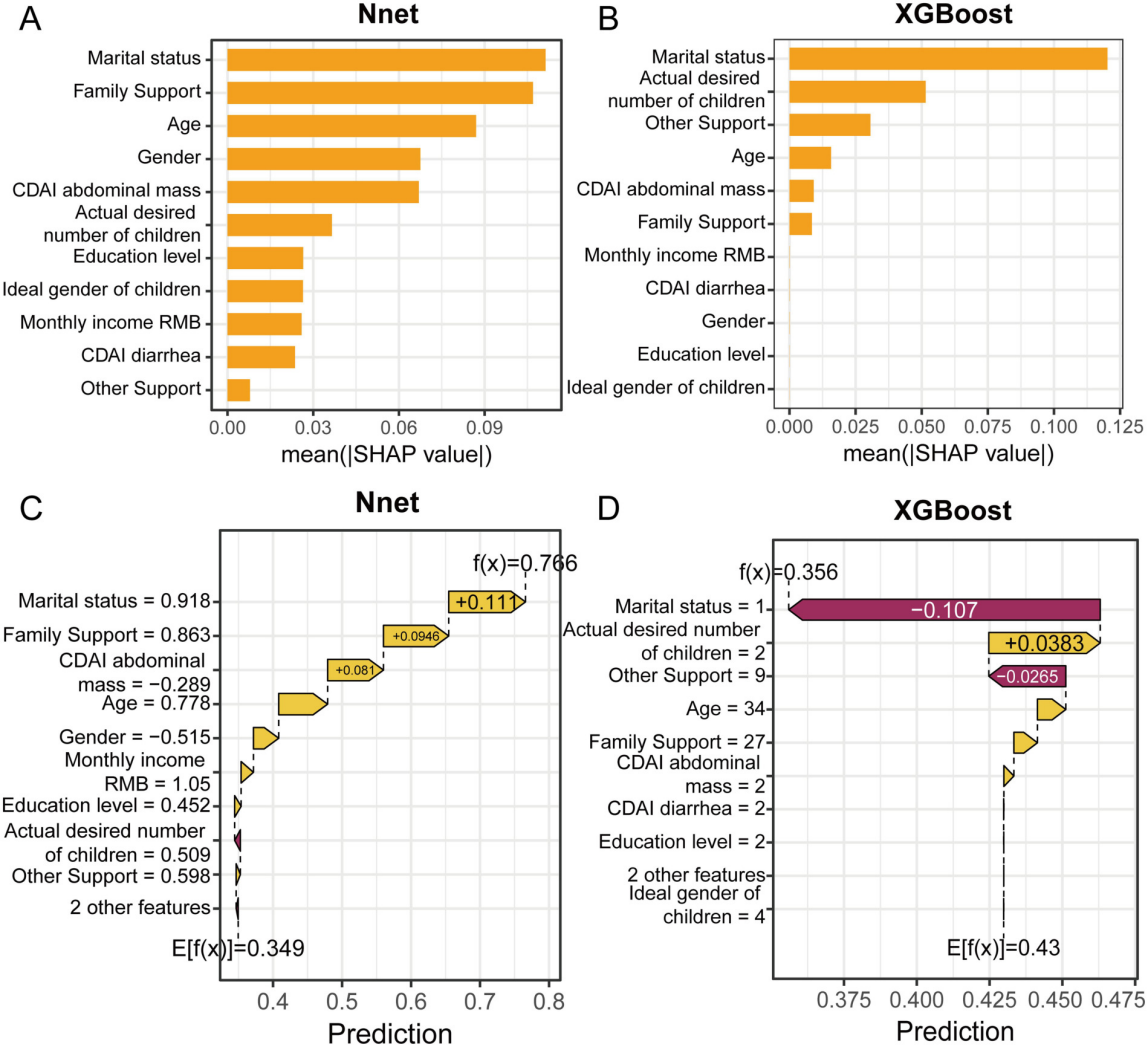
SHAP analysis of neural network and XGBoost models. **(A)** Feature importance ranking plot for the Neural Network model. **(B)** Feature importance ranking plot for the XGBoost model. **(C)** Waterfall plot for patients with high fertility intentions, showing that being married, having strong family support, and experiencing mild disease symptoms increase fertility intentions in patients with Crohn's disease. **(D)** Waterfall plot for patients with low fertility intentions, high lighting factors that reduce fertility intentions, including lack of a spouse, disinterest in having children, and reduced support.

We employed SHAP to interpret the two best-performing and clinically concordant models—the neural network and XGBoost. Figure 3A displays the SHAP feature importance plot for the neural network model, ranked by the impact of each variable on reproductive intention. The results identified Marital status, Family support, and Age as the three most influential variables. Figure 3C presents the SHAP waterfall plot for the neural network model, illustrating the contribution of each variable in a representative patient with high reproductive intention. For instance, Marital status and Family support emerged as the two strongest positive contributors.

Similarly, Figure 3B shows the SHAP feature importance plot for the XGBoost model, where Marital status, Actual desired number of children, and Other support were ranked as the top three predictors. The corresponding SHAP waterfall plot for a patient with low reproductive intention (Figure 3D) revealed that Marital status and Actual desired number of children were the most significant factors associated with reduced reproductive intention.

### 3.5 Marital status and desired number of children as key predictors of fertility intention

To further explore determinants of fertility intention among CD patients, we conducted both univariate and multivariate logistic regression analyses.

In univariate analysis, the following factors were significantly associated with fertility intention (*p* < 0.1): Age (*p* < 0.001), Marital status (*p* < 0.001), Ideal gender of children (*p* = 0.033), Actual desired number of children (*p* < 0.001), Actual desired gender of children (*p* = 0.058), Family Support (*p* < 0.001), Friend Support (*p* = 0.029), Other Support (*p* = 0.001), Comprehensibility (*p* = 0.041), Manageability (*p* = 0.061), and Total Social Support (*p* = 0.001) (Table 3). Total Social Support was excluded from further analysis due to multicollinearity.

**Table 3:** The results of the logistic regression analysis for fertility intention.

	Univariate logistic regression analysis			Multivariable logistic regression analysis		
Variable	OR	95% CI	*P* value	OR	95% CI	*P* value
Age	1.087	1.045–1.130	<0.001	0.989	0.929–1.053	0.730
Gender	0.68	0.369–1.252	0.216			
BMI, kg/m²	1.009	0.971–1.047	0.656			
Education level	1.349	0.698–2.606	0.373			
Marital status			<0.001			0.007
Never married	Ref	Ref	Ref	Ref	Ref	Ref
Married	5.077	2.986–8.631	0.000	4.636	1.996–10.77	0.000
Divorced	1	0.101–9.881	1.000	0.606	0.052–7.056	0.689
Widowed	4.85E + 09	0-Inf	0.999	4.4E + 09	0-Inf	0.999
Other	0	0-Inf	1.000	0	0-Inf	1.000
Monthly income, RMB			0.337			
<5,000	Ref	Ref	Ref			
5,000–10,000	1.545	0.718–3.326	0.266			
>10,000	1.786	0.826–3.865	0.141			
Disease duration, years			0.221			
<5	Ref	Ref	Ref			
5–10	1.465	0.827–2.593	0.190			
>10	1.758	0.79–3.912	0.167			
Biologic biosimilar therapies	0.687	0.286–1.653	0.402			
Immunosuppressants	0.942	0.528–1.68	0.838			
Surgical treatment for IBD	1.037	0.64–1.679	0.882			
The number of chronic diseases			0.732			
0	Ref	Ref	Ref			
1	1.215	0.732–2.018	0.451			
≥2	1.23	0.407–3.713	0.714			
CDAI scores	0.977	0.885–1.078	0.645			
Ideal number of children			0.120			
0	Ref	Ref	Ref			
1	1.02E + 09	0-Inf	0.999			
2	1.42E + 09	0-Inf	0.999			
3 or more	3.23E + 09	0-Inf	0.999			
Other	4.52E + 08	0-Inf	0.999			
Ideal gender of children			0.033			0.781
Male	Ref	Ref	Ref	Ref	Ref	Ref
Female	0.635	0.17–2.371	0.500	0.602	0.046–7.936	0.699
Both	1.128	0.328–3.882	0.848	0.921	0.086–9.865	0.946
Other	0.488	0.136–1.752	0.271	0.542	0.045–6.487	0.629
Actual desired number of children			<0.001			0.001
0	Ref	Ref	Ref	Ref	Ref	Ref
1	1.43E + 09	0-Inf	0.998	9.3E + 08	0-Inf	0.998
2	2.23E + 09	0-Inf	0.998	9.2E + 08	0-Inf	0.998
Other	2.51E + 08	0-Inf	0.998	1E + 08	0-Inf	0.998
Actual desired gender of children			0.058			0.401
Male	Ref	Ref	Ref	Ref	Ref	Ref
Female	1.054	0.312–3.563	0.933	2.444	0.215–27.718	0.471
Both	1.6	0.488–5.25	0.438	1.609	0.152–17.018	0.693
Other	0.709	0.215–2.34	0.572	4.317	0.398–46.847	0.229
Influenced by fertility policies	0.952	0.561–1.615	0.855			
Family Support	1.12	1.058–1.185	<0.001	1.079	0.979–1.19	0.127
Friend Support	1.061	1.006–1.119	0.029	0.956	0.861–1.061	0.399
Other Support	1.09	1.034–1.15	0.001	1.087	0.977–1.208	0.124
Comprehensibility	0.948	0.900–0.998	0.041	1.006	0.896–1.129	0.921
Manageability	0.962	0.924–1.002	0.061	1.01	0.922–1.107	0.825
Meaningfulness	1.065	0.971–1.168	0.180			
Total Social Support	1.037	1.016–1.058	0.001			

Data set in bold are statistically significant. OR, odds ratio; CI, confidence interval.

In the multivariate logistic regression model, Marital status and Actual desired number of children were identified as independent predictors of fertility intention, with odds ratios of 4.636 and 9.3 × 10^8^, respectively (*p* < 0.05), indicating a substantial increase in the likelihood of fertility intention associated with these factors (Table 3).

## 4 Discussion

The results of this study indicate that 39.9% of participants explicitly expressed fertility intentions, suggesting that the fertility desire within this group is moderately low. Similar trends were observed among other chronic disease patients. Huang et al. (17) conducted a survey on 223 breast cancer patients who had undergone chemotherapy, with an average fertility intention score of 41.18 (out of a possible 75 points). Additionally, Armuand et al. (39) investigated fertility intentions in 484 young cancer patients aged 18 to 45, finding that 31% of them expressed a desire for fertility.

In this study, we developed and internally validated eight ML models to predict fertility intentions among patients with CD. To our knowledge, this is the first investigation to systematically compare multiple ML algorithms in this context. The candidate predictors used for modeling were grounded in clinical rationale and prior psychosocial frameworks known to influence reproductive decisions. Among the tested models, the neural network and XGBoost algorithms demonstrated superior performance in terms of discrimination, calibration, and clinical utility based on decision curve analysis, with AUC values of 0.718 and 0.710, respectively.

The neural network model exhibited strong predictive capacity, particularly in correctly identifying individuals with high reproductive intention, as evidenced by its sensitivity in the higher probability regions. Notably, this model's calibration curve revealed close alignment with the ideal prediction line in low-risk strata, further supporting its clinical applicability. The XGBoost model showed a similar calibration pattern and delivered favorable net benefits across a range of threshold probabilities, underscoring the robustness and generalizability of tree-based ensemble learning in predicting psychosocial outcomes. These findings reinforce the value of rigorous model comparison and highlight the practical potential of ML for precision decision-making in chronic disease populations.

Interpretability remains a critical consideration in applying ML models to clinical practice, particularly when models are deployed in sensitive domains such as fertility counseling. To address the “black box” nature of ML, we employed SHAP to deconstruct and visualize individual feature contributions within the neural network and XGBoost models. SHAP analysis revealed that marital status, social support, and actual desired number of children were the most influential predictors of fertility intention. These findings are consistent with previous qualitative and quantitative studies in oncology and reproductive health, where social context and partnership dynamics emerged as central drivers of reproductive decisions.

The high SHAP values associated with marital status and family support emphasize their predictive and clinical relevance. In line with Shapley's cooperative game theory framework, these variables contributed disproportionately to model output, indicating that their absence or alteration could significantly shift individual prediction outcomes. Such insight not only enhances the transparency of ML models but also facilitates targeted intervention design. For example, reproductive counseling programs tailored to unmarried individuals or those reporting low levels of perceived support may be more effective in addressing unmet reproductive needs. Furthermore, the application of explainable AI methods such as SHAP offers a pathway for integrating patient-centered variables into risk stratification tools, thus bridging the gap between data-driven analytics and individualized care.

To complement the ML models, we conducted a multivariable logistic regression analysis. The results revealed that marital status and actual desired number of children were independently associated with positive fertility intentions. These findings echoed the top-ranking features identified via SHAP values in the ML models, enhancing the robustness and clinical relevance of our predictive framework. While logistic regression offers interpretability and simplicity, its performance metrics—including an AUC of 0.645 and lower calibration—were inferior to those of the neural network and XGBoost models. This underscores the potential added value of ML in capturing complex, nonlinear interactions between demographic and psychosocial variables in fertility prediction.

Marital status emerged as the most influential predictor of fertility intention in both ML and logistic regression analyses. This finding aligns with established psychosocial theories emphasizing the role of intimate relationships in shaping reproductive goals. Being married typically entails the presence of a stable partner, shared life planning, and mutual commitment, all of which contribute to the formation of a secure environment conducive to parenthood. In our study, patients in stable marital relationships consistently expressed higher levels of fertility intention, underscoring the significance of spousal support as a psychological buffer against disease-related anxieties. Mechanistically, partnership stability may mitigate the psychological burden associated with chronic disease by enhancing emotional resilience and reducing feelings of isolation. According to dyadic coping theory (40), couples who engage in shared coping strategies in the face of chronic illness demonstrate greater psychological adjustment and more aligned future planning—including reproductive decisions.

Additionally, married individuals may experience less perceived stigma regarding their fertility status, which can be particularly salient in cultures where marriage and parenthood are tightly interlinked. From a clinical standpoint, these findings suggest that marital status is not merely a demographic descriptor but a proxy for emotional, social, and logistical resources critical to fertility-related decision-making. Clinicians should consider incorporating relational assessments into fertility counseling frameworks for CD patients, facilitating couple-based interventions that strengthen mutual support and align reproductive goals (41, 42).

The variable “actual desired number of children” demonstrated a strong and independent association with positive fertility intentions. This variable, often overlooked in clinical practice, captures an individual's pragmatic assessment of their childbearing goals under current circumstances. Unlike ideal fertility preferences—which may reflect abstract or culturally influenced aspirations—actual desired fertility represents a rationalized synthesis of personal desire, relational context,and health-related feasibility. Psychologically, this variable reflects the motivational drive embedded in reproductive planning (43). Even among patients facing health challenges, a high actual desired number of children may indicate strong intrinsic fertility motivation, shaped by personal identity, life narrative, and social expectations. In our analysis, individuals with higher fertility expectations were more likely to override disease-related concerns, illustrating that internal value systems can modulate the perceived risks of childbearing. Importantly, the persistence of this desire in the face of chronic illness suggests a potential leverage point for behavioral and educational interventions. Clinicians should explore this dimension as part of fertility discussions, recognizing that it encapsulates not only fertility planning but also broader constructs of self-actualization, family continuity, and social belonging. Tailored interventions that acknowledge and respect patients' fertility expectations may improve shared decision-making and enhance patient satisfaction with reproductive care.

Perceived family support functioned as a significant enabling factor. According to the Buffer Theory of social support (44), robust support networks can mitigate the negative psychological impact of stressors—in this case, the fears associated with parenting while managing a chronic condition. Support may manifest practically (e.g., promised childcare assistance) and emotionally (e.g., reassurance), thereby reducing the perceived burden and risk of parenthood. Our finding that family support was frequently cited as a key promoting factor underscores its role in shaping a supportive ecosystem for potential parenthood. For CD patients, who often worry about their physical capacity and the hereditary risks of IBD, knowing that a reliable support system exists may be crucial for translating a vague desire into a concrete intention.

The predictive relevance of marital status, fertility expectations, and social support underscores the necessity of a multidimensional, patient-centered fertility assessment protocol in IBD care. By integrating psychosocial factors into routine evaluation, clinicians can stratify patients not only based on medical risk but also in terms of reproductive psychosocial readiness. To translate these insights into clinical practice, several targeted interventions are recommended. For instance, couple-based counseling may help unmarried or ambivalent patients clarify their reproductive goals and address relational uncertainties. Likewise, personalized educational programs focusing on disease control and pregnancy safety can be designed for individuals who exhibit high fertility expectations but lack reproductive confidence. Additionally, enhancing social support networks through peer mentorship and family-inclusive care models may strengthen perceived support and foster reproductive agency. Collectively, these strategies highlight the potential of tailored, context-sensitive interventions to improve reproductive decision-making in patients with chronic disease.

In addition, the integration of explainable ML models into clinical workflows—particularlythose utilizing SHAP for transparency—may enable real-time decision support. For instance, fertility intention risk scores could be embedded into electronic health records, helping clinicians identify and address the contextual and cultural nuances underlying fertility intentions in CD populations, especially within collectivist societies where family planning is a shared decision-making domain.

This study has several limitations. First, the relatively small sample size from a single center may limit the generalizability and robustness of the predictive models, despite the use of 10-fold cross-validation. Second, the cross-sectional design precludes causal inference between fertility intentions and associated factors. Third, all participants were recruited from one hospital in China, which may limit applicability to other cultural and healthcare contexts. Fourth, the inclusion of “actual desired number of children” and “ideal gender of children” as predictor variables warrants careful interpretation. While these constructs were measured separately from the binary fertility intention outcome, their conceptual proximity may introduce circularity and inflate model performance. In particular, the high odds ratio for actual desired number of children in the logistic regression and its prominent SHAP importance likely reflect this close relationship. To mitigate this concern, future studies could either exclude such variables from the predictor set or employ causal discovery frameworks to disentangle their roles. Nevertheless, sensitivity analyses (unreported) in which these two variables were removed still yielded consistent top predictors (marital status, family support, age), suggesting that the main findings are not solely driven by these items. Lastly, the reliance on self-reported data introduces the potential for reporting bias. Future multicenter, longitudinal studies with larger and more diverse populations are recommended to enhance external validity and causal inference.

## 5 Conclusion

In conclusion, this study developed and validated interpretable ML models to predict fertility intentions in patients with CD. Among the eight algorithms, neural networks and XGBoost showed the best performance (AUC > 0.71). SHAP analysis revealed marital status, desired number of children, and perceived family support as key predictors, highlighting the central role of psychosocial factors beyond disease activity. These findings provide a foundation for personalized reproductive counseling and underscore the importance of integrating psychosocial assessment into routine CD care.

## Data Availability

The original contributions presented in the study are included in the article/Supplementary Material, further inquiries can be directed to the corresponding author.
